# Clinical Presentation, Natural History, and Therapeutic Approach in Patients with Solitary Fibrous Tumor: A Retrospective Analysis

**DOI:** 10.1155/2020/1385978

**Published:** 2020-03-26

**Authors:** P. Schöffski, I. Timmermans, D. Hompes, M. Stas, F. Sinnaeve, P. De Leyn, W. Coosemans, D. Van Raemdonck, E. Hauben, R. Sciot, P. Clement, O. Bechter, B. Beuselinck, F. J. S. H. Woei-A-Jin, H. Dumez, P. Nafteux, T. Wessels

**Affiliations:** ^1^Department of General Medical Oncology, University Hospitals Leuven, Leuven Cancer Institute, Leuven, Belgium; ^2^Laboratory of Experimental Oncology, Department of Oncology, KU Leuven, Leuven, Belgium; ^3^Department of Surgical Oncology, University Hospitals Leuven, Leuven Cancer Institute, Leuven, Belgium; ^4^Department of Orthopedic Surgery, University Hospitals Leuven, Leuven Cancer Institute, Leuven, Belgium; ^5^Department of Thoracic Surgery, University Hospitals Leuven, Leuven Cancer Institute, Leuven, Belgium; ^6^Department of Pathology, University Hospitals Leuven, Leuven Cancer Institute, Leuven, Belgium

## Abstract

**Background:**

Solitary fibrous tumor (SFT) is a rare variant of soft tissue sarcoma (STS). *Materials and Methods*. We reviewed SFT patients (pts) treated at our institution between 12/1990 and 09/2017.

**Results:**

We identified 94 pts with a median follow-up (mFU) of 4.7 years (range: 0.1–21.53). Primary sites were the chest (33%), abdomen (21.3%), brain (12.8%), and extremities (9.6%); 6.4% of pts presented with synchronous metastasis. Median overall survival (mOS) from the first diagnosis was 56.0 months (m) (0.3–258.3). Doege–Potter syndrome was seen in 2.1% of pts. Primary resection was performed in 86 pts (91.5%). Median progression-free survival was 34.1 m (1.0–157.1), and 43% of pts stayed SFT-free during FU. Local recurrence occurred in 26.7% after a mFU of 35.5 m (1.0–153.8), associated with an OS of 45.1 m (4.7–118.2). Metachronous metastasis occurred in 30.2% after a mFU of 36.0 m (0.1–157.1). OS in metastatic pts was 19.0 m (0.3–149.0). Systemic therapy was given to 26 pts (27.7%) with inoperable/metastatic disease. The most common (57.7%) upfront therapy was doxorubicin, achieving responses in 13.3% of pts with a PFS of 4.8 m (0.4–23.8). In second line, pts were treated with ifosfamide or pazopanib, the latter achieving the highest response rates. Third-line treatment was heterogeneous.

**Conclusion:**

SFT is an orphan malignancy with a highly variable clinical course and a considerable risk of local failure and metachronous metastasis. Surgery is the only curative option; palliative systemic therapy is used in inoperable/metastatic cases but achieves low response rates. The highest response rates are seen with pazopanib in second/third line.

## 1. Introduction

Sarcomas are a heterogeneous group of malignant tumors arising from mesenchymal cells. Solitary fibrous tumor (SFT) is one of the multiple different histologically or genetically defined subtypes of soft tissue sarcoma (STS). According to RARECARE and based on the analysis of a large population of patients treated in 89 European centers, the incidence of SFT is less than 0.1 per 100,000 person-years [1].

The frequency of SFT is less than 2% among all STS cases, which itself accounts for only 1% of all newly diagnosed malignancies, highlighting the orphan character of this very rare sarcoma subtype [2].

A first publication describing 5 clinical cases of SFT was published in 1931 [3]. Several different terms have been used in the past for this disease, e.g., localized fibrous tumor of the pleura, localized mesothelioma, localized fibrous mesothelioma, localized benign fibroma, or submesothelial fibroma. Another synonymous term is hemangiopericytoma (HPC), described first in 1942 and originally thought to be a neoplasm arising from perivascular smooth muscle cells [4].

With the development of immunohistochemistry techniques and sophisticated cytogenetic analyses, it was shown that HPC and SFT have an identical genetic alteration, the NAB2-STAT6 gene fusion. This fusion is a result of a recurrent inversion of the long arm of chromosome 12 (12q13) and results in the expression of NAB2-STAT6 protein, which is a transcription factor [5]. It serves as a driver for tumor growth by activating NAB2 target genes which have an early growth response-binding domain fused to the activation domain of STAT6. Overexpression of NAB2-STAT6 induces proliferation in cultured cells and activates the expression of early growth response-regulated genes [6].

The identification of this fusion gene as a common driver in both diseases suggests identical pathogenesis of these tumors. SFT and the HPC are currently regarded as one biological entity and are preferably called SFT [7].

Initially, SFTs were thought to arise only in the pleural cavity, but other primary sites have been reported in multiple case reports over the last decades. The main extrapleural sites are the abdominal cavity, orbit, upper respiratory tract, and soft tissue [8]. The clinical course of SFT can be very indolent [9]. Symptoms are usually related to the anatomic location of the disease. Common symptoms of pleural SFT are chest pain, cough, and dyspnea [9]. In other locations, SFT usually presents as a painless mass [10].

Very rarely, these tumors are diagnosed because of a specific endocrine syndrome. SFT patients with Doege–Potter syndrome have recurrent, refractory, clinically relevant hypoglycemia due to paraneoplastic secretion of large insulin-like growth factor 2 by the tumor [11]. A case series published in 1981 described hypoglycemia with clinical symptoms in approximately 4% of patients [12].

The radiological diagnosis of SFT is usually based on computer tomography (CT) and/or magnetic resonance imaging (MRI). A common feature is the hypervascularity of the tumor. Even though the radiological presentation of the tumor can suggest the diagnosis of SFT, histologic examination and genetic analysis of tissue are required to establish a definitive diagnosis [13]. A characteristic feature on a CT scan is a well-circumscribed soft tissue mass with mixed attenuation, which is typically displacing rather than invading surrounding structures [14]. On MRI SFTs display low T1 signal intensity and a variable T2 signal [15].

SFTs are known to have unpredictable clinical behavior, and the natural course can vary from a very indolent, localized tumor to a presentation with early metastasis and aggressive systemic spread [7].

A first risk stratification was published in 1989 by England et al. based on the following histopathological criteria: high cellularity and mitotic activity (>4 mitotic figures per 10 high-power microscopic fields (HPF)), cellular pleomorphism, presence of hemorrhage and necrosis [16]. de Perrot et al. suggested a classification for pleural SFT combining morphological and histopathological features, based on the findings that pedunculated pleural tumors (stage 0) only had a 2% recurrence rate, benign sessile tumors (stage I) relapsed in 8% of cases, malignant pedunculated tumors (stage II) had a 14% recurrence rate, and malignant sessile tumors (stage III) had a 63% recurrence rate and 30% mortality. Malignancy was defined as the presence of the following features: high cellularity, cellular pleomorphism, high mitotic count (>4 per 10 HPF), necrosis, or stromal/vascular invasion. This was the basis for a classification system using a scale from 0 to IV, with stage IV defined as the presence of single or multiple synchronous metastases [17]. A more recent classification proposed by Tapias et al. combined and validated the previous prognostic characteristics for pleural SFT to estimate the risk for recurrence. One point each was given for high mitotic activity, hypercellularity, necrosis/hemorrhage, size >10 cm, sessile (as compared with pedunculated) growth, and parietal pleural origin. According to this system, SFTs are classified as high risk for recurrence when three or more points are present. Low risk tumors have very low recurrence rates of 1.3%, 1.3%, and 3.5% at 5, 10, and 15 years, compared with recurrence rates of 13.5%, 17.5%, and 27.9% in high-risk cases. This score may provide guidance in the postoperative surveillance and FU of this rare tumor [18].

A retrospective analysis of patients with primary resectable pleural and extrapleural SFT showed 5- and 10-year recurrence free rates of 74% and 55%, with 5- and 10-year disease-specific survival of 89% and 73% [19]. Patient age, tumor size, and mitotic index predicted both time to metastasis and disease-specific mortality, while the presence of necrosis predicted metastasis only [19]. Another series focusing on extrathoracic SFT showed that these tumors had a poorer prognosis than pleural SFT, with a 5-year survival of 40%. Adverse histopathological findings were hypercellularity, focal cytological atypia, ≥4 mitotic cells per 10 HPF, tumor necrosis, and/or infiltrative margins [20]. Another smaller retrospective series reported that even in the absence of “negative” histopathological findings (mitotic count ≥4/10 HPF, necrosis, and nuclear polymorphism), recurrence can still occur. Relapses after primary surgery were mostly local (70%), but distant metastasis was also reported. The median time to first recurrence after resection was 12 years and mOS after first recurrence 8 years [21].

Given the fact that recurrence can still occur in the absence of poor prognostic histopathological findings, none of the currently available risk stratifications are fully predictive for the clinical course of individual patients.

A retrospective analysis of the use of radiotherapy (RT) suggested a clinically meaningful benefit for RT given with either curative or palliative intent. In that series, patients treated with definitive RT (60 Gy) had an objective response rate (ORR) of 67%, achieved local disease control after 5 years in 81.3% of cases, and had a 5 year OS of 87.5%. In the case of palliative RT (39 Gy), the ORR was 38% with a 5-year local control rate of 62.5% and a 5-year OS of 54.2% [22]. Data on the use of pre- or postoperative therapy (RT and/or systemic treatments) are very limited in this sarcoma subtype, though responses observed in advanced disease clearly support the use of RT in selected patients.

The treatment approach for advanced, inoperable and/or metastatic disease is very empirical, as published recommendations are either extrapolated from the treatment of other types of STS or based on case reports or unicentric case series. Some authors highlight the therapeutic potential of drugs such as dacarbazine, ifosfamide, or pazopanib in SFT, but this is mainly coming from retrospective case collections [23]. There is some evidence supporting the use of oral antiangiogenic agents in SFT. Recently a first prospective, multicenter, single-arm phase 2 trial suggested activity of pazopanib [24], and another phase 2 trial demonstrated antitumor activity of axitinib [25]. Of interest, Park and colleagues already demonstrated the successful use of an angiogenesis inhibitor in 2011, using bevacizumab in combination with temozolomide [26]. This study showed an overall response rate of 79% using Choi criteria [26].

The fact that most historical STS systemic treatment trials pooled patients with different histological subtypes and that SFTs only represented a fraction of patients in such studies contributes to the lack of reliable clinical information and treatment recommendations for this histological subtype. Furthermore, due to the sometimes indolent and unpredictable pattern of SFT, a number of sarcoma trials have systematically excluded patients with SFT or selected patients bearing SFTs with well documented, more aggressive behavior. This, of course, introduces selection bias and makes it very difficult to draw reliable conclusions from such series.

Given the fact that we see a considerable number of patients with SFT and considering the absence of high level evidence from prospective trials for most treatment options, we wanted to evaluate the clinical course, diagnostic process, prognosis, and treatment of patients with SFT, with the intention of providing further guidance for the optimal treatment of patients with this orphan malignancy.

## 2. Materials and Methods

After obtaining approval by the Medical Ethics Committee of the University Hospitals in Leuven for this analysis, we retrospectively reviewed the records of all patients with SFT/HPC who were diagnosed and treated in our institution between 01/12/1990 and 30/09/2017, including patients transferred from other institutions. Demographic, clinical, and treatment data were extracted from our local, sarcoma-specific database (LECTOR). Data were collected in a Microsoft Excel file (Microsoft, USA) and cross-checked with source data in patient files. Missing data were marked as unknown.

PFS was calculated from the day of primary surgery until the confirmation of a local or metastatic recurrence. OS was calculated as the interval between the first diagnosis of the disease until the death of all causes and from time of recurrence until death from all causes and date of censoring was used. Response rates were classified as complete (CR) or partial response (PR), stable disease (SD), or progressive disease (PD) based on investigator assessment in clinical routine. The methodology for response assessment was not standardized, as this is a retrospective series. Data entry is based on responses documented in the patient files. Figures were made using Prism 5 for Windows (GraphPad Software Inc., USA) and diagrams were made using Microsoft Excel and Powerpoint (Microsoft, USA).

## 3. Results

### 3.1. Patient Characteristics and Epidemiology

We identified 94 patients with SFT/HPC. A total of 42 (45%) of them were female and 52 male (55%). The median age at the time of diagnosis was 56 years (16–85). Our first patient was diagnosed in December 1990 and the last patient was diagnosed in June 2017, with a mFU of 4.7 years (0.1–21.5) (Table 1). During this period, a total of 2124 patients with STS were included in our LECTOR database, which means that the prevalence of patients with SFT in our local STS population is about 4.4%.

### 3.2. Clinical Presentation

**Figure 1:**
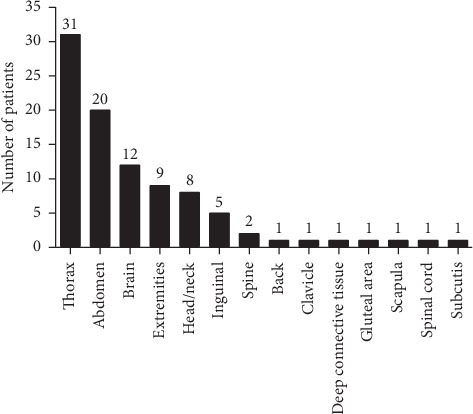
Anatomical localization of the primary solitary fibrous tumor.

The most common primary sites of SFT were chest (33%), abdomen (21.3%), brain (12.8%), extremities (9.6%), head/neck (8.5%), inguinal region (5.3%), spine (2.1%), and other areas (1.1%) (Figure 1).

The symptomatology of SFT patients at first presentation was very variable and mainly dependent on the primary anatomical site of the disease. Patients with thoracic disease usually described a dry cough, dyspnea, or thoracic pain. Patients with abdominal disease mainly had abdominal pain, a palpable mass, swollen abdomen, or unilateral edema. Symptoms of patients with central nervous SFT were headache, limb paresis, or development of paresthesia. Patients with extremity SFT commonly had a painless palpable mass, with few exceptions.

In 22.3% of our patients, the tumor was found incidentally by performing imaging for other medical reasons. In 28.7% of cases, we were not able to identify the reason for the diagnosis, suggesting that the number of incidental cases may even be higher.

Hypoglycemic episodes (Doege–Potter syndrome) as a paraneoplastic symptom associated with SFT were noticed in 2.1% of the patients throughout the course of their disease.

### 3.3. Diagnosis

The initial diagnosis and staging were commonly based on radiological tests, mainly CT and MRI scans. Pathognomonic radiological features of this tumor were not described. In only 7 patients (7.4%) a working diagnosis of SFT was documented in radiological reports prior to establishing a tissue-based diagnosis. The most frequent differential diagnosis on the basis of radiological findings was STS not otherwise specified, with a common reference to leiomyosarcoma as a potential sarcoma subtype.

### 3.4. Prognosis and Recurrence

**Figure 2:**
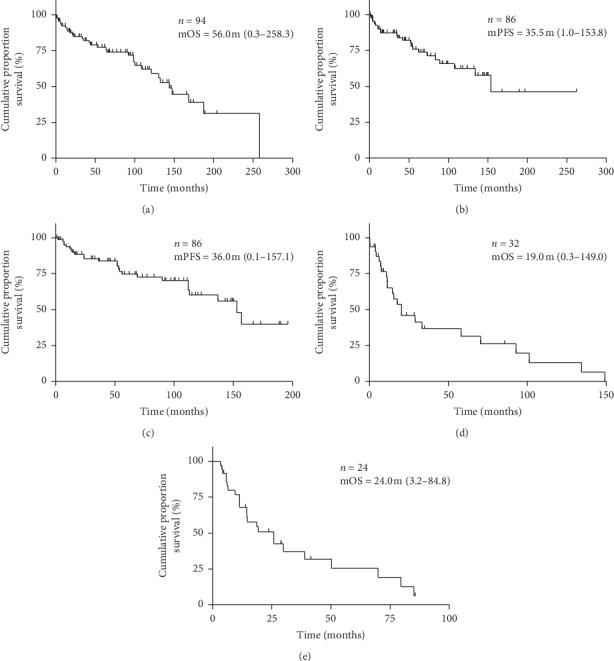
Kaplan–Meier estimate for the following: (a) mOS. (b) Local PFS after primary surgery. (c) Distant PFS after primary surgery. (d) mOS since diagnosis of metastatic disease. (e) mOS since the start of palliative systemic therapy. Survival curves (Kaplan–Meier estimates).

Among the 94 patients with SFT, 4 (4.3%) never received any specific treatment. The mOS of these patients was only 2.8 m (0.2–6.1), likely due to their far advanced stage, age, or comorbidity. Six patients (6.4%) were diagnosed with one or multiple synchronous metastases at diagnosis (Table 1). All others had localized disease. mOS of the 94 patients counted from the first diagnosis was 56.0 m (0.3–258.3) (Figure 2(a)). The 5- and 10-year survival rates were 48.9 and 21.3%, respectively.

A total of 86 patients (91.5%) underwent surgical resection of their primary tumor. Their mPFS was 34.1 m (0.1–153.8). Thirty-seven (43%) stayed SFT-free during FU after primary surgery and were presumably cured (Table 2).

Twenty-three patients (26.7%) who underwent primary surgery developed local recurrence after a median interval of 35.5 m (1.0–153.8) (Table 2); they had either a second resection or systemic therapy. Nine of them (39.1%) developed more than one local relapse.  Figure 2(b) shows the Kaplan–Meier estimate of local PFS after primary resection. The mOS of patients after the first local relapse was 45.1 m (4.7–118.2).

**Figure 3:**
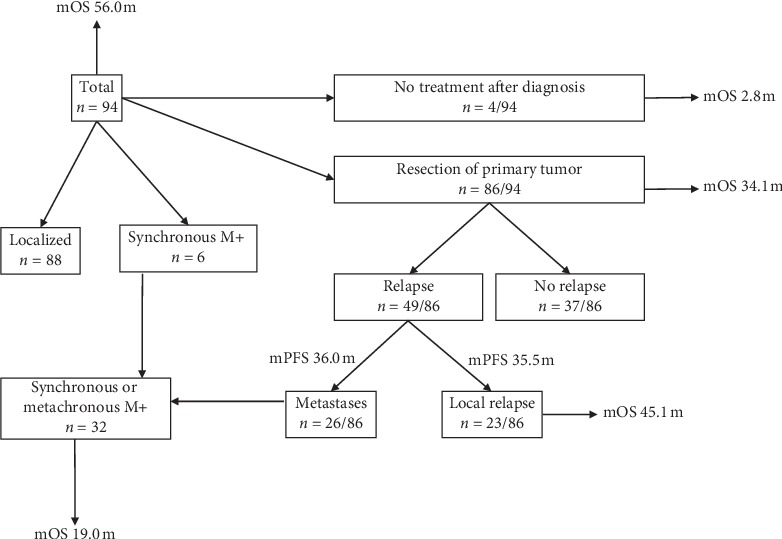
Overview of clinical course of patients included in our retrospective analysis. *n* number of patients, M+: metastasis.

Among 86 patients treated surgically with curative intent, 26 patients (30.2%) developed metastatic disease (Table 2). The median time until the development of metastasis was 36.0 m (0.1–157.1).  Figure 2(c) shows the Kaplan–Meier estimate of distant PFS after primary surgery. The mOS after diagnosis of synchronous or metachronous metastatic disease was 19.0 m (0.3–149.0) (Figure 2(d)).  Figure 3 shows an overview of the clinical course of all patients included.

### 3.5. Local Treatment

Among 86 patients who underwent surgery for the primary tumor, 39 patients (45.5%) were rendered tumor-free after surgery (R0 resection) and 6 (7.0%) had either microscopic (R1) or macroscopic residual disease (R2). Two of the latter patients (33.3%) underwent a wider resection to achieve R0 status. The resection status of 41 patients (47.4%) in the database is not known (Table 2). Twelve had surgery for one or more local recurrences.

A total of 18 patients (20.9%) received postoperative RT, of which 14 after primary resection (16.3%) and 4 after secondary resection (4.6%). Local recurrence after primary surgery and radiotherapy was seen in only one patient (7.1%). Metastatic recurrence after primary surgery and postoperative radiotherapy was seen in 5 cases (35.7%) (Table 2). None of the patients received adjuvant chemotherapy after surgery with curative intent (Table 2).

### 3.6. Systemic Treatment

In the total series of 94 patients, 28 (29.8%) received systemic therapy. Two patients (2.1%) had only neoadjuvant chemotherapy for downsizing the tumor prior to surgery; 24 (25.5%) had palliative systemic therapy; two (2.1%) received both neoadjuvant and palliative systemic during the course of their disease (Table 2). In total 26 patients (27.7%) underwent palliative systemic therapy, their mOS, calculated from the start of systemic treatment, was 24.0 m (3.2–84.4), as illustrated in Figure 2(e).

**Figure 4:**
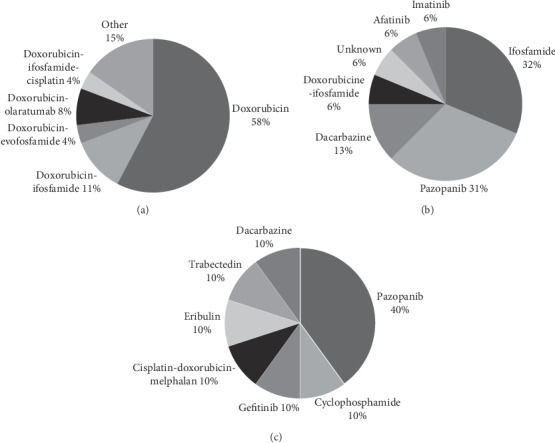
Overview of systemic treatments used in solitary fibrous tumor. (a) First-line systemic treatment (*n* = 26). (b) Second-line systemic treatment (*n* = 16). (c) Third-line systemic treatment (*n* = 10).

A number of different systemic therapies were given (Figure 4). The most frequently used first-line scheme was doxorubicin in 15 patients (57.7%) achieving a partial response (PR) in two patients (13.3%) and stable disease (SD) in 4 cases (26.7%) (Table 3). Other first-line therapies were combinations based on doxorubicin, e.g., doxorubicin/ifosfamide in three patients (11.5%), doxorubicin/olaratumab in two patients (7.7%), doxorubicin/evofosfamide or the combination of doxorubicin, ifosfamide, and cisplatin in one patient each (3.8%) (Figure 4(a)). mPFS with first-line doxorubicin-based therapy was 4.8 m (0.4–23.8).

Only 16 patients received second-line systemic therapy. The most commonly used agents were ifosfamide and pazopanib, both in 5 patients (31.3%) (Figure 4(b)). Of the patients receiving ifosfamide, none showed a response. Of 5 patients receiving pazopanib, three (60%) had a PR (Table 3). Dacarbazine was used as second line in only two patients (12.5%), with no objective response achieved.

Ten patients were exposed to third-line treatment, of whom 4 (40%) received pazopanib. Only one patient showed PR (25%) and two had SD (50%) (Table 3). Other patients in third line received individualized treatments based on gefitinib, dacarbazine, eribulin, cyclophosphamide, cisplatin/doxorubicin/melphalan, or trabectedin (Figure 4(c)).

In total 5 patients continued with fourth-line treatment. Three of them were treated with trabectedin (60%). Two of these patients (66.7%) showed SD after three cycles and remained stable at 6 m (Table 3). Other patients were individually treated with cisplatin or cyclophosphamide (20%). After further progression, only three patients continued systemic treatment. Two received 6 lines of treatment and one even had 9 lines of systemic therapy on an individual basis.

## 4. Conclusions

We present epidemiological data, the clinical presentation, diagnostic process, recurrence rates, and treatment regimens used in SFT based on a single-center retrospective analysis. We used our sarcoma-specific database and electronic records to analyze all SFTs treated over a period of 27 years. For the purpose of this analysis, we combined SFT and HPC as one entity, considering the NAB2-STAT6 fusion in both conditions.

SFT is extremely rare, even in our referral center with specific sarcoma expertise. The prevalence of patients with SFT in our STS population is twice as high as what is described in RARECARE [2], and the number of new sarcoma cases in our academic institution is much higher than in other hospitals in the country. We believe this is due to a selection bias related to the referral character of our institution, which has strong histopathological and genetic expertise in rare sarcomas. In addition, specific multidisciplinary surgical, radiotherapeutic, and oncological skills are present here, together with the availability of clinical trials. More than 4% of all STS seen in our institution are SFTs.

The Doege–Potter syndrome was seen in 2.1% of our SFT patients and was mainly treated symptomatically. This frequency is somewhat lower than what was described in an older retrospective analysis [12]. Paraneoplastic hypoglycemia remains an exceedingly rare complication of this disease and most oncologists will never see this condition during their career. Interestingly, one of our SFT patients diagnosed with recurrent paraneoplastic hypoglycemia and who was treated with pazopanib in combination with local radiotherapy achieving a PR showed a significant decrease in the number of hypoglycemic episodes. This illustrates that efficient systemic treatment of the underlying malignancy could help improve hypoglycemia, similar to other paraneoplastic syndromes in epithelial malignancies.

Even though our series is among the largest reported in the literature, data about PFS and OS are still based on relatively small numbers of patients, and it is very difficult to compare our findings with older case series. Recurrence rates reported in different series have been highly variable and some have used different definitions (pathological, anatomical) and risk classifications. Patients with the extrapleural disease tend to do worse than patients with pleural disease, and this can be the reason why our data shows survival rates somewhere in between the currently available data [18–20]. Our progression and survival analysis shows a broad range outcome, confirming the very variable and unpredictable course of SFT. While describing survival outcomes from this retrospective series, we want to highlight here that we purposely did not use any scale for risk classification, due to missing data in this 27-year series, but also because of the lack of reliable, standardized pathological criteria for the according analysis and the finding that currently available risk stratifications are not fully predictive for the clinical course of SFT.

Only 16.3% of our primary resected patients received adjuvant radiotherapy. The local recurrence rate (7.1%) after adjuvant radiotherapy was lower than the local recurrence rate in the whole population (26.7%). This supports the use of adjuvant radiotherapy to prevent local recurrence. Not unexpectedly, adjuvant radiotherapy failed to result in a decrease of metachronous metastatic disease (35.7% vs. 30.2%).

None of our patients received adjuvant systemic therapy. In analogy with other STS subtypes, the use of adjuvant chemotherapy should probably be discussed individually with patients at high risk of recurrence; ideally, such patients should be included in prospective clinical trials [27]. The very variable course of SFT makes joint decision making on postoperative treatments very difficult.

If we compare SFT with other STS subtypes, our data support the concept that SFT clinically has a more unpredictable behavior and histological grading alone does not predict the risk of developing the metastatic disease [28].

A significant proportion of patients (29.8%) in our series required systemic therapy, as they developed inoperable local relapses and/or distant metastasis. Systemic treatment choices were extrapolated from the experience with anticancer agents in other STS subtypes. In the majority of our patients first-line palliation was based on anthracyclines, with a preference for single-agent doxorubicin (57.7%), reflecting the experience that metastatic SFT is poorly sensitive to more aggressive chemotherapy and given the known absence of overall survival advantage with the use of anthracycline-based combinations in STS. mPFS of doxorubicin-based therapy in first line in our series was 4.8 m, which is in accordance with the data of another retrospective first-line study in SFT patients which showed a mPFS of 4.0 m [23]. Interestingly these Kaplan–Meier estimates are also in line with results with doxorubicin in unselected STS populations, where the most relevant prospective series resulted in a mPFS of 4.6 m [29].

The second-line treatment in our analysis was usually based on ifosfamide (31.3%) or the angiogenesis inhibitor pazopanib (31.3%). Our analysis suggests that higher response rates can be achieved with pazopanib, which is also perceived as a much more tolerable treatment option by most patients.

Our observation provides a potential rationale for the earlier use of an angiogenesis inhibitor in SFT. Our data are in line with key findings of a very recent Mediterranean phase 2 trial with pazopanib in SFT, which suggested that PRs and SD can be achieved in 51% and 26% of patients [24]. Pazopanib is approved in many countries for nonadipocytic STS, which includes SFT, in patients after prior exposure to chemotherapy [30].

Based on our data and in accordance with large prospective trials combining multiple subtypes of STS [29], we believe that the use of aggressive combination therapy in metastatic SFT cannot be recommended. We advocate the sequential use of currently available systemic single agents, but recognize the relative absence of data regarding the best sequence of potential systemic treatments. Currently, available data are mainly based on single-center phase 2 trials with highly selected patients and some retrospective analyses.

Given the retrospective nature of our study, it is difficult to draw definitive conclusions. There is a clear need for more prospective work in this field of oncology, with a disease-specific focus on SFT. We have seen some interesting multicenter data on the use of pazopanib, axitinib, and radiation therapy in the literature more recently [22, 24, 25]. Given the epidemiology of SFT, it remains difficult (if not impossible) to perform larger scale, ideally randomized trials to standardize the treatment of this orphan disease, but the sarcoma community should clearly join forces to do more prospective, SFT-specific trials.

## Figures and Tables

**Table 1:** Patient characteristics.

	*n* (%)
Patients	94
Median age (years)	56
Sex	
Male	52 (55)
Female	42 (45)
Synchronous metastasis	6 (6.4)

**Table 2:** Treatment outcome, recurrence status, and adjuvant- and systemic therapy.

	*n* (%)
Patients	94
Primary resection	86 (91.5)
R0	39 (45.5)
R1	6 (7)
Rx	41 (39.1)
Recurrence	
Local recurrence	23 (26.7)
Metachronous metastasis	26 (30.2)
No recurrence	37 (43.0)
Adjuvant radiotherapy	14 (16.3)
Local recurrence	1 (7.1)
Metastatic recurrence	5 (35.7)
Adjuvant systemic therapy	0 (0)

Systemic therapy	28 (29.8)
Neoadjuvant	2 (2.1)
Palliative	24 (25.5)
Neoadjuvant and palliative	2 (2.1)

Treatments applied in 94 patients with SFT. R0: complete resection. R1: microscopic residual disease. Rx: unknown resection status.

**Table 3:** Response rates.

	CR	PR	SD	PD	NE
First line					
Doxorubicine (*n* = 15)	0/15	2/15	4/15	7/15	2/15
Consecutive lines					
Pazopanib (*n* = 9)	0/9	4/9	2/9	2/9	1/9
Ifosfamide (*n* = 5)	0/5	0/5	0/5	4/5	1/5
Dacarbazine (*n* = 2)	0/2	0/2	1/2	1/2	0/2
Trabectedin (*n* = 3)	0/3	0/3	2/3	1/3	0/3

CR: complete response PR: partial response, SD: stable disease, PD: progressive disease, NE: not evaluable or available. Patients treated with pazopanib (*n* = 9) in second (*n* = 5) and third line (*n* = 4) were pooled for this analysis. Best response per single-agent systemic therapy and per treatment line according to treating physician.

## Data Availability

The data used to support the findings of this study have been deposited in the LECTOR repository.

## Additional Points

*Key Message*. Solitary fibrous tumor, an orphan malignancy with unpredictable, highly variable clinical course, has a low incidence of distant spread at diagnosis but considerable risk of local failure and metachronous metastasis. Surgery is the only curative option at diagnosis and in case of resectable relapse/metastatic disease. Palliative systemic therapy is used in inoperable, metastatic cases, with the highest response rates observed with angiogenesis inhibition. *Implications for Practice*. Solitary fibrous tumor is a very rare soft tissue malignancy with a variable, unpredictable clinical course. Less than half of pts are cured by surgery; others develop inoperable disease or distant metastasis. The disease is sensitive to radio- and chemotherapy. Response rates to chemotherapy are as low as in other types of soft tissue sarcoma. Drugs directed against the tumor's vasculature and angiogenesis inhibitors such as pazopanib offer an active and well-tolerated alternative that should be studied in prospective trials.
