# Corrigendum to “Clinical Presentation, Natural History, and Therapeutic Approach in Patients with Solitary Fibrous Tumor: A Retrospective Analysis”

**DOI:** 10.1155/2021/4653987

**Published:** 2021-01-29

**Authors:** P. Schöffski, I. Timmermans, D. Hompes, M. Stas, Veerle Boecxstaens, F. Sinnaeve, P. De Leyn, W. Coosemans, D. Van Raemdonck, E. Hauben, R. Sciot, P. Clement, O. Bechter, B. Beuselinck, F. J. S. H. Woei-A-Jin, H. Dumez, P. Nafteux, T. Wessels

**Affiliations:** ^1^Department of General Medical Oncology, University Hospitals Leuven, Leuven Cancer Institute, Leuven, Belgium; ^2^Laboratory of Experimental Oncology, Department of Oncology, KU Leuven, Leuven, Belgium; ^3^Department of Surgical Oncology, University Hospitals Leuven, Leuven Cancer Institute, Leuven, Belgium; ^4^Department of Orthopedic Surgery, University Hospitals Leuven, Leuven Cancer Institute, Leuven, Belgium; ^5^Department of Thoracic Surgery, University Hospitals Leuven, Leuven Cancer Institute, Leuven, Belgium; ^6^Department of Pathology, University Hospitals Leuven, Leuven Cancer Institute, Leuven, Belgium

In the article titled “Clinical Presentation, Natural History, and Therapeutic Approach in Patients with Solitary Fibrous Tumor: A Retrospective Analysis” [1], a co-author's name was missed in the author list. The corrected author list is as above.
